# Postural Control and Neuromuscular Activation in 11–13-Year-Old Athletic Boy Swimmers

**DOI:** 10.3390/children11070863

**Published:** 2024-07-16

**Authors:** Rym Baccouch, Ghada Jouira, Cristina Ioana Alexe, Dragoș Ioan Tohănean, Dan Iulian Alexe

**Affiliations:** 1 Research Laboratory Education, Motricité, Sport et Santé (EM2S) LR19JS01, High Institute of Sport and Physical Education of Sfax, University of Sfax, Sfax 3029, Tunisia; baccoucherim@yahoo.fr (R.B.); jouiraghada0825@gmail.com (G.J.); 2 Department of Physical Education and Sports Performance, “Vasile Alecsandri” University of Bacău, 600115 Bacau, Romania; 3 Departament of Motric Performance, “Transilvania” University of Brașov, 500036 Brașov, Romania; 4 Department of Physical and Occupational Therapy, “Vasile Alecsandri” University of Bacău, 600115 Bacău, Romania; alexedaniulian@ub.ro

**Keywords:** center of pressure, electromyography, anteroposterior, vision, swimming practice

## Abstract

Objective: This study compared postural control and neuromuscular activation in athletic swimmers (A-S) and non-athletic swimmers (N-A-S) in older children. Methods: Ten A-S and ten N-A-S underwent assessments of center of pressure (CoP) parameters under static and dynamic surfaces in two directions (dynamic mediolateral (DML) and dynamic anteroposterior (DAP)) in eyes-open (EO) and eyes-closed (EC) conditions, and electromyography (EMG) parameters under DAP and DML directions in EO and EC conditions. Results: Results showed that A-S demonstrated significantly superior postural control (*p* < 0.05), with smaller CoP area and lower CoP mean velocity compared with N-A-S, particularly in static with EC, DAP with EO and EC, and DML with EO conditions. A-S exhibited significantly larger neuromuscular activation amplitudes (*p* < 0.05), especially in the AP direction. Conclusions: These findings suggested that athletic swimming training may enhance postural control and neuromuscular activation in 11–13-year-old children, emphasizing the potential benefits of incorporating swimming exercises in these children.

## 1. Introduction

Postural control, defined by the ability to keep the body’s center of gravity balanced over its base of support when stationary, is essential for all human movement. It is indispensable for sustaining an upright posture and ensuring balance during various daily life [1] and athletic [2] activities. Achieving effective postural control requires the integration and coordination of somatosensory, visual, and vestibular inputs through the central nervous system [3]. Additionally, it involves executing motor control processes to generate musculoskeletal responses that keep the body within its stability limits [4].

In the developmental lifespan, children typically exhibit less developed balance control compared with adults [5,6,7,8]. In addition, older children aged between 9 and 12 years experience significant transformations in body size, shape, and composition, characterized by a noticeable growth spurt [9,10]. It has been suggested that these physiological changes may influence postural control [11]. Furthermore, this developmental phase introduces distinct neurohormonal alterations, which may lead to temporary reductions in postural control due to the complex interaction between hormonal shifts and the ongoing maturation of physiological systems [12,13]. Additionally, there is evidence indicating a progressive decline in balance-system functioning during this transition period, a phenomenon that persists until approximately age 13 [14]. Regular physical activity is crucial during this period to promote the development and refinement of postural control [15,16], as participation in physical activities is consistently associated with improved postural control [17].

Research has widely demonstrated that adult athletes demonstrate greater postural skills than sedentary individuals [18,19,20,21,22] and they acquire new balance-control skills, which possibly differ depending on the discipline practiced [18,23,24]. Sports practice facilitates the adaptation and mastery of postural strategies specific to the demands of each sport. Postural strategy refers to how the body organizes itself spatially and temporally, and it involves the coordination of body segments and the sequence of muscle activation [25,26]. It also encompasses the integration of sensory inputs, highlighting the significance of different sensory information and neural pathways in regulating posture [25]. Indeed, the strategy used for postural control is influenced by a combination of physiological, biomechanical, and environmental factors [27]. The environmental context, such as swimming, significantly influences the use of sensory information [28,29].

Swimming differs from most other sports because of its unique characteristics, including the prone position, immersion in water, and the use of propulsive forces against a constantly fluctuating environment [30]. In aquatic environments, the traditional visual references for horizontality are altered or partially absent due to immersion [31,32]. As a result, swimmers must heavily compensate with enhanced proprioceptive information to maintain postural control [33,34]. Proprioceptive feedback from muscles, tendons, and joints becomes paramount, informing the brain about the body’s position and movement in the water [33]. This reliance on proprioception helps swimmers to adjust their movements and maintain balance, and ensures efficient propulsion despite the challenging aquatic environment [34]. In addition, swimming involves deliberate movements aimed at maximizing speed through coordinated movements of the arms, legs, and body to overcome water resistance [35]. It represents a distinct form of exercise compared with traditional exercises that involve weight bearing. This primarily involves engaging the extremities and body in tasks performed underwater [28,36,37]. Occurring in a near-zero-gravity environment due to the buoyancy of the water, swimming provides a comprehensive full-body workout targeting various muscle groups, and promoting strength, endurance, and cardiovascular fitness [38,39,40,41]. Although studies have suggested that participation in swimming activities could enhance postural balance capabilities in older individuals [28,35,42,43], the impact of swimming training on children’s postural control remains uncertain. While infants exposed to regular baby swimming programs exhibited enhanced motor performance [29], regular participation in standard swimming education among adolescents showed minimal effect on postural defects [44]. The relationship between swimming and postural control in older children aged between 11 and 13 years old remains underexplored in the scientific literature.

Additionally, beyond its impact on postural control, swimming also influences neuromuscular activation patterns [45]. The unique demands of swimming, such as the need for coordinated movements in an aquatic environment, require precise neuromuscular control to achieve effective propulsion and maintain postural control [28]. Studies have shown that swimming uses a wide range of muscle groups, including superficial and deep muscles [46,47], to generate propulsive forces and stabilize the body against water resistance. Although the effects of swimming on neuromuscular activation have been studied primarily in adults, more research is needed to understand how these adaptations develop in older children.

Examining the impact of swimming on postural control and neuromuscular activation in older children is especially significant due to the unique sensory variations present in aquatic environments. Swimming presents challenges such as unstable surfaces and vision variability due to the refractive properties of water, which can significantly impact the use of sensory information for postural control and neuromuscular activation, requiring swimmers to rely more on proprioceptive feedback and less on visual input to maintain balance and control movements. Therefore, studying the impact of swimming on postural control and neuromuscular activation under different sensory-integration conditions in children aged 11–13 years is essential for several reasons. First, understanding how swimming affects these aspects during a crucial period of development can inform the design of interventions aimed at optimizing children’s postural health and neuromuscular development. Second, given the variety of sensory challenges presented by the aquatic environment, such as impaired proprioception and vision, exploring the effects of swimming under different sensory-integration conditions may provide valuable insight into how sensory inputs influence postural control and neuromuscular activation in this population. Therefore, the current study aimed to evaluate postural control and neuromuscular activation in older children aged 11–13 years engaged in regular swimming activities compared to their sedentary counterparts, and to investigate how different sensory-integration conditions, such as impaired proprioception and visual input, affect postural control and neuromuscular activation in older children engaged in swimming activities. It was hypothesized that older children who regularly participated in swimming activities would display improved postural control and more efficient neuromuscular activation patterns compared with sedentary children of the same age. Additionally, under conditions of impaired sensory integration, such as reduced visual feedback or altered proprioceptive input, swimmers were expected to demonstrate greater adaptability in terms of postural control and neuromuscular activation compared with sedentary children.

## 2. Materials and Methods

### 2.1. Participants

The sample size was determined using G∗Power 3.1 software (Franz Faul, University of Kiel, Kiel, Germany) [48]. With the statistical probability value (α) set at 0.05, a statistical power of 0.80, and a non-sphericity correction of 1, the effect size for this study was estimated at 0.4 based on previous literature [49] and authors’ discussions. This calculation resulted in an initial sample size requirement of 12 participants.

The recruitment process was carried out in three phases. Initially, we screened two groups of boys, totaling 25 children, from the databases of the two nearest schools. The first group comprised 14 athletic swimmers (A-S) who regularly practiced swimming at the municipal swimming pool. They had between 5 and 6 years of experience, training four times a week for 90 min per session. The second group consisted of 11 sedentary boys (non-athletic swimmers (N-A-S)) who did not engage in regular physical activity, defined as structured or consistent physical exercise beyond daily routines [50], and all indicated low physical activity levels based on their responses to nine questions from the Physical Activity Questionnaire for Older Children (PAQ-C) [51,52]. The PAQ-C is recognized as a valid tool for older children to recall their activities over the past seven days to assess their physical activity levels, and is scored between 1 and 5 [51,52,53]. A pediatrician, applying Tanner’s (1962) criteria, categorized them as pre-pubertal (stage 1). In the second stage, we selected 21 children from the screened groups—10 from the A-S group and 11 from the N-A-S group—who met the inclusion criteria. These criteria included middle socio-economic status based on parents’ income, education, and occupation; absence of physical or mental conditions; no history of locomotor surgeries; no respiratory dysfunctions; no visual or auditory impairments; no cardiovascular or metabolic disorders; and no injury in the past 12 months, as verified from the school’s enrollment records. In the third stage, one child was excluded due to absence during the familiarization session, resulting in 20 participants for the study: 10 from the A-S group and 10 from the N-A-S group (Table 1) Once normality was confirmed, the independent t-test indicated non-significant differences in age, height, mass, and foot size between the groups (Table 1). Foot size was specifically considered to address potential anthropometric variations that might affect postural control [54].

Following a detailed explanation of procedures, including the potential risks and benefits involved, children provided their assent, while their parents or legal guardians provided written informed consent.

### 2.2. Study Design

This study adopted a cross-sectional comparative design to evaluate and compare postural control and neuromuscular activation between two groups of older children aged 11 to 13 years: athletic swimmers (A-S) and non-athletic swimmers (N-A-S). Postural control was assessed using a series of static and dynamic conditions. In the static (S) condition, participants maintained a standing position on a stabilometric platform for a duration of 51.2 s. During the dynamic mediolateral (DML) and dynamic anteroposterior (DAP) conditions, lasting 25.6 s each, participants stood on a seesaw that oscillated in the ML and AP directions, respectively. Electromyography (EMG) signals were recorded during the maintenance of a bipedal stance in the two dynamic postural conditions (DAP and DML) under EO and EC conditions. The evaluations were conducted in the morning by the same evaluators to maintain consistency. The study consisted of three laboratory visits spaced at least 2 days apart. During the initial visit, participants familiarized themselves with the equipment and procedures. The second and third visits involved the testing sessions, where evaluations of postural control and EMG were conducted.

### 2.3. Measurements

#### 2.3.1. Postural Control

The force platform measurements used in this study were both valid and reliable, ensuring accurate and consistent assessment of postural control parameters [55,56,57]. Participants were instructed to stand as steadily as possible on a force platform with arms resting comfortably at their sides, feet positioned 30° apart and heels 5 cm apart. A plastic device ensured consistent foot placement. They completed three standing conditions: static (S) for 51.2 s, and dynamic ML (DML) and AP (DAP) on a seesaw (radius 55 cm, arrow 6 cm) for 25.6 s each, altering platform orientation [58]. Six experimental conditions were tested: S-EO, S-EC, DAP-EO, DAP-EC, DML-EO, and DML-EC. Participants in the EO condition were instructed to fix their gaze straight ahead at a white cross positioned on the wall 2 m away at eye level. In the EC condition, participants were directed to maintain a horizontal gaze straight ahead. There were three trials in each experimental condition, with invalidation occurring only in cases of complete loss of balance. The best trial of the three was selected for analysis to represent postural behavior. To prevent fatigue, a 1 min rest interval was included between trials, during which participants sat comfortably in a chair. Data collection began after participants assumed the required posture on the platform, stabilized their balance, and signaled readiness to start. Throughout the recording session, the experimenter remained nearby for safety, refraining from physical contact or issuing additional instructions, ensuring consistent posture maintenance. In this study, two main parameters were used to assess participants’ postural control: the center of pressure (CoP) area (CoP_area_), which represents the surface area of the confidence ellipse derived from 90% of successive CoP positions, and the mean CoP velocity (CoP_Vm_), calculated as the sum of CoP displacement divided by the sampling time. These metrics are widely acknowledged for their reliability in evaluating postural control efficiency [59]. Lower values of these parameters indicate better postural control.

#### 2.3.2. EMG

The EMG measurements used in this study were valid and reliable [60,61]. EMG signals were recorded during bipedal stance in dynamic postural conditions (DAP and DML). Following the European Recommendations for Surface Electromyography [62], electrodes were placed longitudinally on the gastrocnemius medialis (GM) and tibialis anterior (TA) muscles of the right leg. The GM electrodes were positioned on the muscle’s most prominent bulge, while those for TA were located at 1/3 distance from the fibula on a line between the fibula’s tip and the medial malleolus. A reference electrode was affixed to the olecranon of the right elbow. To ensure optimal electrode–skin contact, skin impedance was kept below 2 kΩ through shaving, abrasion, and cleaning with an alcohol–ether–acetone solution before placement. Test contractions were performed to verify RMS noise levels below 12 μV prior to exercise. EMG signals were preamplified using a differential amplifier (Bagnoli-4 EMG System; Delsys Inc., Natick, MA, USA) with a CMRR of 92 dB, input impedance over 1015 Ω, and a gain of 1000. Signals were filtered between 20 and 450 Hz, digitized at 1000 Hz with 16-bit accuracy, and stored for analysis using EMGworks 3.0 Delsys Analysis software.

The root mean square (RMS) values, reflecting muscle contraction amplitude, were averaged over 30 s segments for TA and GM muscles, per subject and condition, to assess neuromuscular activation at the ankle joint [63,64]. Higher RMS values indicate better neuromuscular activation.

### 2.4. Statistical Analysis

Data were analyzed using SPSS 25.0 (Statistical Package for the Social Sciences Inc., Chicago, IL, USA). Normality was confirmed using the Shapiro–Wilk test. For the CoP measurements, a three-way ANOVA (2 Group × 3 Surface × 2 Vision) with repeated measures was used to determine the effect of group and/or postural condition and/or vision on the dependent variables: CoP_Vm_ and CoP_area_. The Group factor had two levels: A-S and N-A-S. The Surface factor had three levels: S, DAP, and DML. The Vision factor had two levels: EO and EC. The RMS of the postural muscles at the ankle joint was analyzed using a three-way ANOVA (2 Group × 2 Direction × 2 Vision). The Direction factor had two levels: ML and AP. The partial eta squared (ηp^2^) formula was used to calculate the effect sizes for main effects and interactions (small: 0.01 < ηp^2^ < 0.06; moderate: 0.06 < ηp^2^ < 0.14; large: ηp^2^ > 0.14), while Cohen’s d was computed for pairwise differences (trivial: d < 0.2; small: 0.2 ≤ d < 0.5; moderate: 0.5 ≤ d < 0.8; large: d ≥ 0.8) [65]. Bonferroni adjustment was applied for multiple comparisons, with a significance level set at *p* < 0.05. Mean values and *p*-values were reported with a 95% confidence interval.

## 3. Results

### 3.1. CoP Parameters

Regarding the CoP_area_, significant main effects for Group, Vision, and Surface were identified in the ANOVA results. Additionally, significant interactions were observed for Vision × Group, Surface × Group, Surface × Vision, and Surface × Group × Vision (Table 2). Similarly, for CoP_Vm_, significant main effects were observed for Group, Vision, and Surface, along with significant Vision × Group, Surface× Group, Surface × Vision, and Surface × Group × Vision interactions (Table 2).

**Figure 1:**
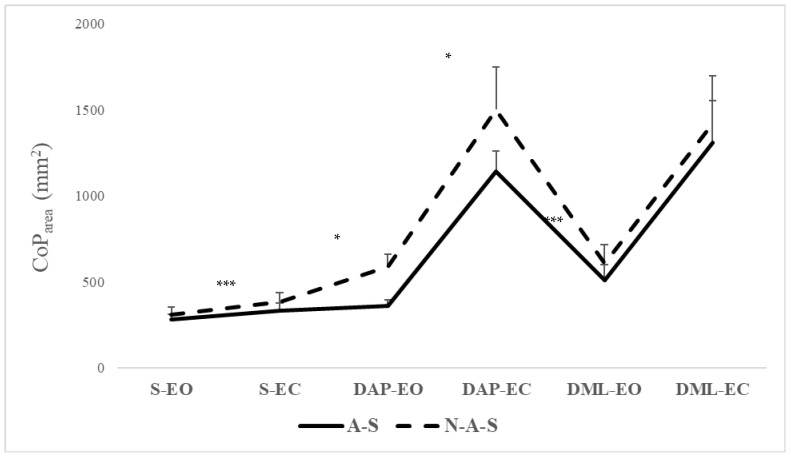
CoP_area_ values in A-S and N-A-S under different surfaces and directional conditions in EO and EC conditions. A-S: athletic swimmers, N-A-S: non athletic swimmers, CoP: center of pressure, S: static, DAP: dynamic anteroposterior, DML: dynamic mediolateral, EO: eyes open, EC: eyes closed. * Significant differences between A-S and N-A-S at *p* < 0.05; *** Significant differences between A-S and N-A-S at *p* < 0.001.

**Figure 2:**
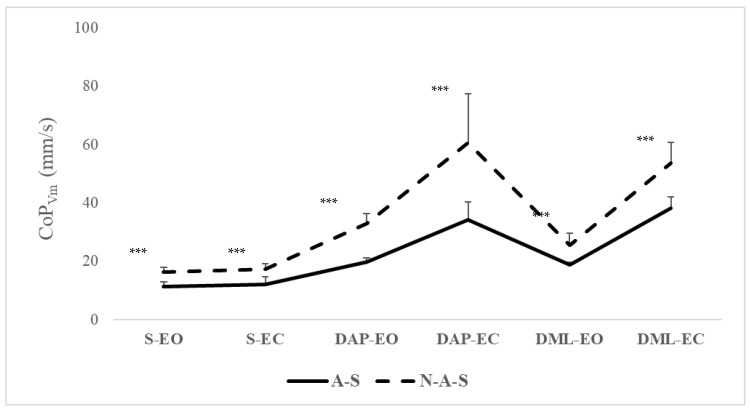
CoP_Vm_ values in A-S and N-A-S under different surfaces and directional conditions in EO and EC conditions. A-S: athletic swimmers, N-A-S: non athletic swimmers, CoP: center of pressure, CoP_Vm_: center of pressure mean velocity, S: static, DAP: dynamic anteroposterior, DML: dynamic mediolateral, EO: eyes open, EC: eyes closed. *** Significant differences between A-S and N-A-S at *p* < 0.001.

Post hoc analysis revealed that the A-S group showed significantly smaller CoP_area_ (*p* < 0.05) and lower CoP_Vm_ (*p* < 0.001) compared with the N-A-S group in the S-EC condition, as well as in the DAP-EO, DAP-EC, and DML-EO conditions (Figure 1 and Figure 2). However, no significant differences were observed in the S-EO or DML-EC conditions (Table 3). The switch from EO to EC significantly increased the CoP_area_ values (*p* < 0.001) for both groups in the static, DML, and DAP conditions. This transition led to increased CoP_Vm_ values in the same conditions (*p* < 0.001), except in DAP for both groups and in DML only for the A-S group. Post hoc analysis indicated significantly smaller CoP_area_ (*p* < 0.001) and lower CoP_Vm_ (*p* < 0.001) in the S condition compared with the two dynamic conditions (DAP and DML) in both EO and EC conditions. The A-S group exhibited significantly smaller CoP_area_ (*p* = 0.009) in the DAP condition compared with the DML condition under EO (Figure 1 and Figure 2).

### 3.2. EMG Parameter

Regarding the RMS, significant main effects were observed for Group, Vision, and Direction. Significant interaction effects were found for Direction × Group. No significant interactions were observed for Vision × Group, Direction × Vision, or Direction × Group × Vision (Table 2).

**Figure 3:**
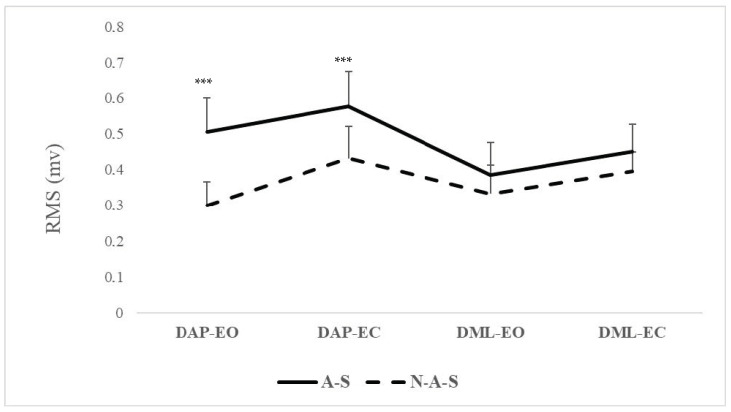
RMS values in A-S and N-A-S under directional conditions in EO and EC conditions. A-S: athletic swimmers, N-A-S: non athletic swimmers, RMS: root mean square, S: static, DAP: dynamic anteroposterior, DML: dynamic mediolateral, EO: eyes open, EC: eyes closed. *** Significant differences between A-S and N-A-S at *p* < 0.001.

Post hoc analysis revealed a significantly larger amplitude of contraction in the A-S group compared with the N-A-S group only in the AP direction under both EO and EC conditions (*p* < 0.001) (Table 3, Figure 3). The switch from EO to EC significantly increased the RMS values for both the A-S (*p* = 0.03) and N-A-S (*p* = 0.002) groups, only in the AP direction.

## 4. Discussion

The results showed that the A-S group exhibited significantly smaller values of CoP_area_ and lower CoP_Vm_ compared with the N-A-S group under specific conditions. Particularly, significant differences were observed in the S-EC condition and in dynamic conditions (DAP-EO, DAP-EC, and DML-EO), where the A-S group demonstrated reduced CoP_area_ and CoP_Vm_ values.

Swimming requires precise coordination of limb movements and body positioning to reduce water resistance and improve propulsion [66]. This coordination involves the synchronization of the upper and lower limbs to effectively propel the body through the water [67]. Our findings suggest that swimmers in the A-S group may have developed specialized postural strategies to maintain stability in aquatic environments, involving adjustments in neuromuscular activation and proprioceptive feedback. It has been proposed that water’s buoyancy could enhance postural control by mitigating the effects of gravitational forces [68]. Indeed, buoyancy, the fundamental principle of swimming, provides unique benefits for exercise and movement. It works against gravity, effectively reducing stress on joints and facilitating multidimensional movements [69]. This feature allows individuals to participate in exercises that would otherwise be difficult on the floor, enhancing postural control and adaptability, even for children with limited mobility [68]. Additionally, this reduction in gravitational impact is beneficial because it reduces the load on the joints, making movements easier and smoother [68]. Furthermore, immersion in water can relieve muscle tension and spasms [70], thereby optimizing the effectiveness of exercise sessions. In addition, the resistance encountered when moving against the flow of water stimulates muscle growth and strength, which contributes to improved overall fitness over time [38,39,40,41,71]. As well, swimming training exposes individuals to dynamic balance challenges while moving forward or adjusting their swimming posture in water, amidst unstable conditions such as water turbulence and waves [72]. It has been shown that training in unstable aquatic environments, such as those where the water is turbulent, can result in changes in muscle activity compared with stable conditions [73]. Subsequently, all these adaptations may contribute to enhanced postural control. In addition, A-S exhibited greater postural control in the AP direction under EO and EC conditions. This observation suggests that athletes rely less on visual cues than non-athletes to maintain postural control in this specific direction. This phenomenon could be attributed to their increased ability to compensate for the absence of visual input by relying more on other sensory feedback mechanisms [24], such as proprioceptive and vestibular signals. The aquatic environment requires swimmers to continuously adapt their postural control strategies due to the variability in visual references and the need for heightened proprioceptive feedback. Additionally, it has been indicated that prolonged swimming training enhances plantar cutaneous sensitivity thresholds [74,75]. Consequently, swimming training may exert a significant influence on postural control, particularly given the role of the plantar sole as a “dynamometric map” for bipedal balance regulation [75,76]. It is important to note that the results showed no significant differences between the A-S and N-A-S groups in the S-EO or DML-EC conditions. In fact, in the S-EO condition, where individuals maintain balance with the EO on a static surface, the task may indeed be relatively easier than in more difficult conditions. This simplicity could potentially minimize observable differences between groups. Given the simplicity of such a condition, it was found difficult to distinguish between their postural control [77]. On the other hand, in the DML-EC condition, individuals must maintain a dynamic balance in ML direction with EC, which is difficult and poses a greater challenge. Such a condition further challenges the proprioceptive and vestibular systems, as visual input is removed [78]. Therefore, the A-S and N-A-S groups struggled more equally in this condition.

The results showed that the increase in CoP_area_ values under CE compared with OE conditions in all conditions for both groups could be attributed to the loss of visual feedback, which plays a crucial role in maintaining balance by providing information about orientation and position of the body in space [79]. Without visual input, individuals must rely more on proprioceptive and vestibular signals to control posture, leading to increased postural sway [79]. In swimmers, the need to compensate for the lack of stable visual references in water enhances their reliance on proprioceptive feedback, allowing them to maintain balance and stability despite the challenging environment. This result was observed in CoP_Vm_ in the same conditions except in the DAP condition for both groups and in the DML condition only for the A-S group. Previous studies have highlighted the importance of proprioceptive feedback, vestibular signals, and neuromuscular responses in adapting to the challenges of maintaining stability along the AP axis during dynamic movements [80,81,82]. Interestingly, this appears to persist even when vision is removed, indicating a consistent adaptation regardless of visual input. Furthermore, the differential response observed in the DML condition for the A-S group suggests that swimming training may confer specific adaptations in postural-control mechanisms, particularly in dynamic tasks involving ML movements. It is possible that the unique demands of swimming, which involve precise coordination of limb movements and trunk stability in a dynamic aquatic environment, result in specialized adaptations and specific physiological and neuromuscular adjustments [21] that are particularly relevant for maintaining balance during DML movements. Future studies are needed to confirm these findings and further elucidate the mechanisms underlying the observed variations in postural responses across different task conditions.

Surface had a significant effect on all postural sway parameters, indicating greater postural control on a static surface compared with a dynamic surface for both groups and in visual conditions. Challenges encountered in maintaining dynamic conditions arise mainly from biomechanical constraints and sensory characteristics. Previous studies have indicated that during quiet standing on a stable, flat platform, individuals often display slight sway, where the body oscillates around the ankle joint axis, resembling movements of an inverted pendulum [83,84]. Alternatively, when standing on a seesaw surface, individuals position their center of gravity directly above the contact point between the seesaw and the floor [84,85]. It has been indicated that as task demands increase, the motion of the CoP also increases [86], suggesting that balance control is influenced by the task’s level of difficulty [87]. Comparing both directions, the A-S group exhibited significantly smaller CoP_area_ in the DAP condition compared with the DML condition under EO. This result indicated that athletic swimming training may confer a greater enhancement in AP stability compared with ML stability. A possible explanation for this finding is related to the nature of swimming movements. Indeed, swimming involves coordinated movements of the arms and legs to propel the body forward through the water [88]. These movements occur primarily along the AP axis, as swimmers push against water resistance to move forward [88]. Therefore, A-S may develop specialized adaptations to improve stability and control, specifically in the AP direction.

Neuromuscular activation, as indicated by RMS values of postural muscles at the ankle joint, revealed that A-S group exhibited larger muscle-response amplitudes compared with N-A-S. This enhancement in neuromuscular function appears to be linked to the effects of athletic swimming training. Indeed, the endurance–resistance training regimen commonly practiced by A-S is known to induce specific adaptations in muscle properties. Endurance training can strengthen muscles such as the gastrocnemius [89], which plays a crucial role in postural control [90], whereas high-resistance strength training can promote hypertrophy of muscle fibers [91]. In addition, swimming exercises are known to develop the muscular core [46]. The resistive nature of water, with its density being significantly greater than that of air, provides a conducive environment for muscle power development without undue strain on the joints [92]. Moreover, consistent participation in swimming training may lead to specialized adaptations in the nervous system, enhancing the transmission of neural impulses [28,93]. These adaptations may contribute to larger contraction amplitudes during the execution of postural skills [94]. Our findings align with a previous study comparing sedentary individuals with physically active adults, which also demonstrated changes in neuromuscular characteristics, particularly in postural muscles involved in balance control [95]. The observed significant interaction between group and direction further highlights the superior neuromuscular activation in the A-S group, particularly in the AP direction in both visual conditions. Thus, for older children, participation in athletic swimming may lead to improvements in neuromuscular activation of ankle-joint postural muscles, particularly in the AP direction. In addition, the study results showed that the RMS values increased under the EC compared with the EO condition for both the A-S and N-A-S groups, only in the AP direction. This implies that the absence of visual input similarly enhanced muscle-response amplitudes for both groups in these conditions. The increase in RMS values can indeed be attributed to the increased need for neuromuscular recruitment to maintain postural stability when visual input is absent. When individuals close their eyes, they lose the visual references essential to maintaining balance, leading them to rely more on proprioception and vestibular input. The increased RMS values observed in the AP direction indicate an increased demand for muscle activation to manage AP sway. This increased muscle activation is likely due to the need for enhanced proprioceptive feedback to effectively adjust body position and prevent falls [96,97,98]. It has been established that swimming could increase the percentage of ankle strategy which is usually used to keep balance especially in the vision-removal condition [28]. This strategy involves controlling postural balance through ankle adjustments rather than through the hip [99]. Individuals with higher static postural control typically regulate balance more at the ankle [99,100], while those with lower postural control rely more on the hip [101]. This supports our findings that swimming training enhances the use of the ankle strategy used to maintain balance, especially in conditions where visual cues are absent, highlighting the critical role of proprioceptive feedback in maintaining stability. These results suggest that for older children aiming to improve postural control, exercises emphasizing AP stability should be prioritized. Among these exercises, athletic swimming training appears to be a potential option.

There were some limitations in the present study that should be taken into account when interpreting the results. This study did not consider gender-specific effects due to the significant hormonal changes occurring in both boys and girls within the 11 to 13 year age group. Future studies could expand on our findings by incorporating a larger, more diverse sample that includes an equal representation of male and female participants. In addition, it is necessary to compare the postural control abilities of A-S with practitioners of different sports in order to tell parents which sports are the most effective in developing specific postural control in children. Moreover, by selecting participants predominantly at Tanner stage I, we ensured they were at a similar stage of pubertal development, minimizing variability in maturation among participants and reducing the potential influence of pubertal differences on the study outcomes. Future studies should explore a range of Tanner stages to gain a more comprehensive understanding of how pubertal maturation affects postural control. Additionally, future studies should consider the maturity and age of participants when they began athletic swimming training, as these factors could potentially influence balance performance. Furthermore, our study utilized an unstable platform to assess neuromuscular activation, where the velocity of postural perturbations was not standardized. Therefore, future research should employ motor-driven surface translations to ensure consistent perturbation velocities across all participants. It is important to note that, while our research enhances our understanding of postural-control mechanisms developed through swimming training, particularly in aquatic environments, further investigation is needed to determine the extent to which these skills generalize to terrestrial conditions. Future studies should delve into the underlying mechanisms of skill transfer from water to land and assess how aquatic training enhances balance and postural control across different environments.

### Practical Implications

The study findings have practical implications for athletic trainers and individuals seeking to improve postural control and neuromuscular activation, particularly in older children. Athletic trainers can incorporate swimming drills into training programs to address AP stability deficits, particularly in dynamic and visually challenging settings. Additionally, the study highlighted the potential benefits of swimming practice for overall postural control, suggesting that it may be an effective modality for improving balance and reducing the risk of falls for older children who participate in swimming practice or consider swimming part of their routine.

## 5. Conclusions

The study showed that the A-S group exhibited significantly smaller CoP_area_ and lower CoP_Vm_ compared with the N-A-S group under various conditions, indicating improved postural control in A-S. Additionally, A-S demonstrated greater neuromuscular activation amplitudes, particularly in the AP direction, suggesting specific adaptations induced by swimming training. These results highlighted the potential benefits of swimming training to improve postural control and reduce the risk of falls in children aged 11 to 13 years, who may experience temporary deficits in postural control due to the complex interplay between hormonal fluctuations and continued physiological maturation. Incorporating swimming exercises into training programs can improve postural control and neuromuscular function, particularly focusing on AP stability.

## Figures and Tables

**Table 1:** Participants’ characteristics.

	A-S (N = 10)		N-A-S (N = 10)		Degrees of Freedom	Independent-*t* Test	Cohen’s d
	Mean (SD)	Coefficient of Variation	Mean (SD)	Coefficient of Variation			
Age (years)	12.10 ± 0.31	2.56	11.9 ± 0.33	2.77	18	NS	0.3
Height (cm)	147 ± 2.9	1.97	146 ± 3.2	2.19	18	NS	0.4
Mass (kg)	38.01 ± 1.7	4.47	37.00 ± 2.5	6.75	18	NS	0.3
Foot size (cm)	38.02 ± 2.2	3.15	38.00 ± 2.00	5.26	18	NS	0.1

Abbreviations: A-S: athletic swimmers, N-A-S: non-athletic swimmers; NS: non-significant.

**Table 2:** ANOVA results.

	F	Degrees of Freedom	*p*	ηp²
***CoP_area_***				
Group	22.27	1.18	<0.001	0.55
Vision	452.12	1.18	<0.001	0.96
Vision × Group	1.045	1.18	=0.32	-
Surface	392.87	2.17	<0.001	0.97
Surface × Group	14.35	2.17	<0.001	0.62
Surface × Vision	166.42	2.17	<0.001	0.95
Surface × Group × Vision	0.63	2.17	0.544	-
***CoP_Vm_***				
Group	75.61	1.18	<0.001	0.80
Vision	193.48	1.18	<0.001	0.91
Vision × Group	11.56	1.18	=0.003	0.39
Surface	259.63	2.17	<0.001	0.96
Surface × Group	11.69	2.17	<0.001	0.57
Surface × Vision	123.23	2.17	<0.001	0.93
Surface × Group × Vision	4.98	2.17	=0.020	0.37
**RMS**				
Group	28.97	1.18	=0.001	0.61
Vision	46.87	1.18	<0.001	0.72
Vision × Group	0.86	1.18	=0.35	-
Direction	7.93	1.18	=0.011	0.30
Direction × Group	9.34	1.18	=0.007	0.34
Direction × Vision	0.63	1.18	=0.43	-
Direction × Group × Vision	0.37	1.18	=0.54	-

**Table 3:** Comparative analysis of center of pressure and root mean square parameters in athletic and non-athletic swimmers under different surfaces and directional conditions in eyes-open and eyes-closed conditions.

	A-S		N-A-S		*p*-Value	95% CI	d
	Mean (SD)	95% CI	Mean (SD)	95% CI			
**CoP_area_**							
S-EO	281.55 (31.30)	259.15 to 303.94	307.5 (44.24)	275.86 to 339.15	=0.147	−10.04 to 61.96	-
S-EC	330.59 (44.80)	298.53 to 362.64	382.37 (53.39)	344.17 to 420.57	<0.001	178.15 to 282.690	−1.05
DAP-EO	359.73 (33.94)	335.44 to 384.01	590.15 (70.97)	539.37 to 640.92	=0.038	6.22 to 194.99	−4.14
DAP-EC	1142.80 (116.95)	1059.15 to 1226.48	1500.68 (248.79)	1322.72 to 1678.63	=0.034	5.47 to 98.09	−1.84
DML-EO	507.15 (93.28)	440.41 to 573.88	607.79 (107.13)	531.11 to 684.40	<0.001	175.23 to 540.49	−1.00
DML-EC	1309.99 (240.90)	1137.65 to 1482.32	1422.45 (275.19)	1225.58 to 1619.13	=0.340	−130.53 to 355.45	-
**CoP_Vm_**							
S-EO	11.48 (1.46)	10.43 to 12.53	16.22 (1.66)	15.02 to 17.44	<0.001	3.26 to 6.20	−3.03
S-EC	12.08 (2.68)	10.16 to 14.00	17.38 (1.82)	16.07 to 18.68	<0.001	11.06 to 15.70	−2.31
DAP-EO	19.71 (1.52)	18.68 to 20.80	33.10 (3.14)	30.84 to 35.35	<0.001	3.64 to 9.53	−5.42
DAP-EC	34.21 (6.01)	29.90 to 38.51	60.54 (16.85)	48.48 to 72.59	<0.001	3.13 to 7.45	−2.02
DML-EO	18.82 (0.93)	18.16 to 19.49	25.42 (4.33)	22.32 to 28.51	<0.001	14.43 to 38.21	−2.10
DML-EC	38.19 (3.80)	35.47 to 40.91	53.59 (7.09)	48.51 to 58.66	<0.001	10.04 to 20.74	−2.70
**RMS**							
DAP-EO	0.50 (0.09)	0.43 to 0.57	0.30 (0.06)	0.25 to 0.34	<0.001	−0.28 to −1.27	2.61
DAP-EC	0.57 (0.09)	0.50 to 0.64	0.41 (0.10)	0.34 to 0.49	<0.001	−0.25 to −0.67	1.68
DML-EO	0.38 (0.09)	0.32 to 0.45	0.33 (0.08)	0.27 to 0.39	=0.182	−0.13 to 0.27	-
DML-EC	0.45 (0.07)	0.39 to 0.50	0.39 (0.05)	0.35 to 0.50	=0.073	−0.11 to −0.006	-

Abbreviations: A-S: athletic swimmers, N-A-S: non athletic swimmers, CoP: center of pressure, CoP_Vm_: center of pressure mean velocity, RMS: root mean square, S: static, DAP: dynamic anteroposterior, DML: dynamic mediolateral, EO: eyes open, EC: eyes closed.

## Data Availability

The data that support the findings of this study are available on request from the corresponding author. The data are not publicly available due to privacy or ethical restrictions.
